# Transcervical, Transabdominal and Transvaginal Chorionic Villus Sampling for Prenatal Diagnosis in Zagreb, Croatia: A Prospective Single-Operator Study on 5500 Cases

**DOI:** 10.3390/diagnostics15212750

**Published:** 2025-10-30

**Authors:** Petra Podobnik, Tomislav Meštrović, Mario Podobnik, Ivan Bertović-Žunec, Igor Lončar, Kristian Kurdija, Dženis Jelčić, Zlata Srebreniković, Slava Podobnik-Šarkanji

**Affiliations:** 1 Department of Obstetrics and Gynaecology, Podobnik Special Hospital, Ul. Sveti Duh 112, 10000 Zagreb, Croatia; petra@podobnik.hr (P.P.); ivan.bertovic.zunec@podobnik.hr (I.B.-Ž.); igor.loncar@podobnik.hr (I.L.); kkurdija@podobnik.hr (K.K.); djelcic@podobnik.hr (D.J.); zlata@podobnik.hr (Z.S.); slava@podobnik.hr (S.P.-Š.); 2 DA VINCI Polyclinic, Petrovaradinska Ulica 110, 10000 Zagreb, Croatia; 3 University Centre Varaždin, University North, 42000 Varaždin, Croatia

**Keywords:** TA-CVS (transabdominal chorionic villus sampling), TC-CVS (transcervical chorionic villus sampling), TV-CVS (transvaginal chorionic villus sampling), prenatal diagnosis, first-trimester screening, fetal loss rate, diagnostic yield, perinatal safety

## Abstract

**Background/Objectives**: Chorionic villus sampling (CVS) is a pivotal diagnostic tool for early prenatal detection of chromosomal and genetic abnormalities; however, the safety and diagnostic efficacy of different CVS approaches remain a subject of clinical interest. This monocentric study compares transcervical (TC-CVS), transabdominal (TA-CVS) and transvaginal (TV-CVS) techniques, focusing on procedure-related fetal loss and diagnostic yield. **Methods**: In this 15-year, single-operator prospective study, a total of 5500 women underwent CVS between 10 and 14 weeks of gestation at a single center. Sampling was performed via TA-CVS (*n* = 4500), TC-CVS (*n* = 850), or TV-CVS (*n* = 150). Outcomes assessed included fetal loss rates, sample adequacy, early complications and hemodynamic changes measured by Doppler ultrasound. A *p*-value < 0.05 (two-tailed) was considered statistically significant. **Results**: Spontaneous abortion rates were significantly lower following TA-CVS (0.18%; 8/4500) compared to TC-CVS (0.6%; 5/850) and TV-CVS (1.3%; 2/150) (χ^2^ = 24.56, *p* < 0.001). Post hoc pairwise analysis showed significantly lower fetal loss in TA-CVS compared to TC-CVS, but not between TA-CVS and TV-CVS. Cytogenetic abnormalities were detected in 220 cases (4.0%), and clinically significant copy number variants (CNVs) were confirmed in fetuses with major structural malformations. Five-year follow-up showed no diagnosed intellectual disability among assessed children. Optimal tissue weight (10–20 mg) was more frequent with TA-CVS (66.7%) than TC-CVS (35.3%) or TV-CVS (36.7%) (χ^2^ = 350.92, *p* < 0.001). In a Doppler subset (*n* = 400), uterine, spiral, and interplacental artery PI changes were non-significant; the umbilical (*p* = 0.032) and middle cerebral arteries (*p* < 0.001) showed transient PI reductions after sampling. **Conclusions**: Transabdominal CVS demonstrated the most favorable balance of safety and diagnostic quality, suggesting it should be the preferred first-line technique in early prenatal diagnosis. Standardized technique and operator training remain critical to optimize outcomes.

## 1. Introduction

Chorionic villus sampling (CVS) has become an increasingly common method for first-trimester prenatal diagnosis of genetic and chromosomal disorders. Recognized as both effective and relatively safe for early detection, CVS offers a viable alternative to second-trimester amniocentesis. Comparative studies conducted in the United States and Canada report high sampling success rates for CVS (98.2%), closely approaching those of amniocentesis (99.5%). However, these studies also indicate a slightly higher risk of fetal loss associated with CVS—by approximately 0.8% in U.S. studies and 0.6% in Canadian cohorts [1,2,3,4,5,6,7,8,9,10].

Multiple centers and randomized trials have reported that spontaneous abortion rates following CVS are comparable to those observed after amniocentesis [1,2,6,8,10]. While establishing a direct causal link between fetal loss and CVS remains challenging—primarily due to confounding factors such as maternal age and reproductive history—certain procedural elements have been identified as influencing the risk of miscarriage [10,11,12,13,14,15,16,17,18]. These include the type of CVS approach used, the design of the cannula, the number of sampling attempts, as well as the experience level of the operator [10,11,12,13,14,15,16,17,18]. CVS is typically performed using one of three approaches: transcervical access with a catheter or forceps, transabdominal insertion of a spinal needle into the placenta under continuous ultrasound guidance, or transvaginal sampling guided by endovaginal ultrasound to obtain chorionic villi.

Compared to transcervical and transvaginal approaches, transabdominal chorionic villus sampling (TA-CVS) offers several potential advantages. Unlike the transcervical (TC-CVS) and transvaginal (TV-CVS) techniques which involve vaginal manipulation and the introduction of a cannula or needle through the bacteria-colonized endocervix with limited maneuverability, TA-CVS provides more direct and flexible access to the placenta, minimizes the risk of infection, and avoids any vaginal instrumentation. Globally, over 500,000 early CVS procedures have been conducted to date. Reported fetal loss rates vary considerably across individual centers, ranging from 0.2% to 7% [1,2,3,4,5,6,7,8,9,10,19,20,21,22,23]. It has to be emphasized that lower fetal loss rates are typically observed in high-volume centers, particularly those with experience in managing over 1000 procedures [16,17,18,21,22,23,24,25,26,27,28].

The main question we wanted to answer was: what is the excess risk of pregnancy loss and other complications attributable to CVS by sampling route (TA-CVS, TC-CVS, TV-CVS) within standardized first-trimester windows, compared with contemporaneous, eligibility-matched controls who declined invasive testing. We also assessed route-specific cytogenetic yields (including targeted genetic analyses when major ultrasound anomalies were present) and 5-year neurodevelopment (diagnosed intellectual disability). The study also examined potential confounders such as maternal age, sampling gestational week, route of access and number of attempts, with the objective of identifying best-practice parameters that could inform clinical guidelines and reduce procedure-related risks.

## 2. Materials and Methods

Between January 2008 and May 2023, TC-CVS, TA-CVS and TV-CVS were performed in 5500 singleton pregnancies by a single operator (M.P.) as a prospective study. Women aged 37 or older at their expected due date, as well as those with other clinical indications for prenatal diagnosis, were informed and offered participation in the study. Eligibility criteria required a confirmed viable singleton pregnancy under 14 weeks of gestation, along with the ability to attend four scheduled ultrasound examinations—initially between 10 and 14 weeks, followed by scans at 16–20 weeks, 28–32 weeks, and after 36 weeks of gestation. The patients were also re-examined immediately if they experienced bleeding, amniotic fluid leakage or abdominal discomfort. Participants who declined enrollment were instead offered alternative diagnostic procedures, such as placental biopsy or amniocentesis. Exclusion criteria included non-viable pregnancy detected at the initial ultrasound, the presence of multiple gestations, pre-existing maternal Rh(D) alloimmunization (clinically significant antibody titers), and/or untreated cervical infections. A follow-up anomaly scan was performed for all participants between 18 and 22 weeks of gestation to assess for additional fetal abnormalities, followed by routine monitoring ultrasounds at 28–30 weeks and again after 35 weeks. The study was conducted in accordance with the Declaration of Helsinki, and approved by the Ethics Committee of the Podobnik Special Hospital (protocol code 1–2; date: 21 June 2008). An informed consent (both verbal and written) was obtained from all participating women.

All study participants were instructed to notify the hospital of any occurrences of spontaneous or induced pregnancy loss to take into account previous fetal losses. Also, women remaining in the study were telephoned at 20–24 weeks gestation to obtain information about complications or problems with their pregnancies. In cases where a spontaneous abortion occurred before 28 weeks of gestation, the relevant fetal loss documentation was completed. Follow-up was carried out until after 42 weeks to obtain information about the delivery. For all 5500 participants, only essential details were collected regarding the delivery—specifically, these were gestational age, weight at birth and any visibly apparent congenital anomalies. The cohort comprised all consecutive eligible cases with no caps or quotas, counts are database-derived exacts, and indications were assigned by a predefined primary-indication hierarchy to avoid double counting. Controls were consecutive, contemporaneous pregnancies referred through the same pathways and eligible for the same indications as the CVS cohort, but who declined invasive testing (invasive diagnostic intervention) after counselling (primarily for personal or religious reasons), with indications coded by the same primary-indication hierarchy.

We evaluated the effectiveness of transabdominal chorionic villus sampling in relation to the transcervical and transvaginal approaches, and investigated the factors associated with an increased risk of spontaneous miscarriage. We compared miscarriage rates between pregnancies that underwent chorionic villus sampling (the study group) and those that did not involve any invasive procedures (the control group). The procedure-related risk of pregnancy loss was determined by calculating the difference in miscarriage rates between these two cohorts, based on the assumption that baseline maternal and pregnancy characteristics were comparable between the control group and the study group—except that the control group did not undergo any invasive prenatal procedures. Data on pregnancy outcomes and newborn infants were collected from delivering hospitals; these records included details of any complications during pregnancy or childbirth, as well as data on the newborn and placenta. In addition, pediatric follow-up reports on intellectual development were requested five years post-delivery for 4400 participants.

At the outset of the study, only transcervical chorionic villus sampling (TC-CVS) was routinely performed. TV-CVS was introduced selectively in cases where TC-CVS or TA-CVS could not be technically conducted—typically due to a posteriorly located placenta in the context of a retroverted and retroflexed uterus. To assess the relative safety and performance of the three approaches, we compared early procedure-related complications, including vaginal bleeding, infections, early spontaneous miscarriages, elective terminations, abnormal cytogenetic findings, and overall fetal loss occurring before 28 weeks of gestation. Maternal age, parity, indications for prenatal diagnosis, and gestational age at the time of sampling were comparable across all groups.

**Figure 1:**
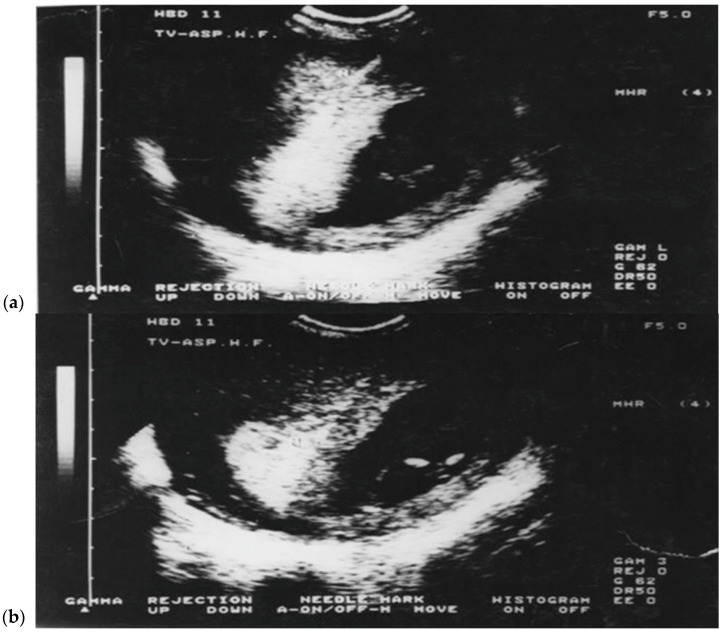
(**a**,**b**) Transvaginal chorionic villus sampling (TV-CVS) performed at 11 weeks of gestation. The biopsy route in TV-CVS is notably longer compared to the transabdominal approach (TA-CVS). Source: Authors.

TC-CVS was conducted with the patient in the lithotomy position. Aspirations were performed at 10 to 13.6 weeks of gestational age. To perform the aspiration, we used a 26 cm long Portex catheter or a 24 cm long Holzgreve-Angiomed catheter, with continuous real-time ultrasonographic guidance provided using a Voluson 8 or 10 scanner, with a 5–9 MHz transducer. The catheter was inserted via cervix into *chorion frondosum*, rotating the catheter to aspirate the tissue into a 20 mL plastic syringe prefilled with 5 mL of culture medium. Amounts of 1 to 25 mg of *chorion frondosum* tissues were obtained and the modified short term-culture (overnight), with modification, was used in all cases for karyotyping [19,20,21,22,24,25,26,27,29,30,31,32,33]. On the other hand, TA-CVS was performed with the patient in the supine position, utilizing a single a 20-gauge-90 mm spinal needle. After disinfecting the anterior abdominal wall and ensuring the patient’s bladder was empty, the needle was inserted under continuous ultrasound guidance into the central region of the *chorion frondosum*. The insertion angle was gradually adjusted by 20 to 40 degrees as needed, and tissue was aspirated under negative pressure into a 20 mL syringe prefilled with the designated culture medium. Furthermore, TV-CVS was performed with the patient in the lithotomy position. To perform aspiration, we used a 35 cm long 20-gauge needle with continuous real-time ultrasonic guidance provided with a 5–9 MHz vaginal transducer with a guide needle. The needle was inserted through the fornix (anterior or posterior) into *chorion frondosum* (Figure 1a,b) and 20 mL syringe was used to aspirate the tissue by combining repeated strong suction and slow backward and forward movement of the needle tip. After two failed passes, for all three techniques the women were offered another CVS session a week later. All 5500 early CVS procedures were performed by a single operator (M.P.).

**Figure 2:**
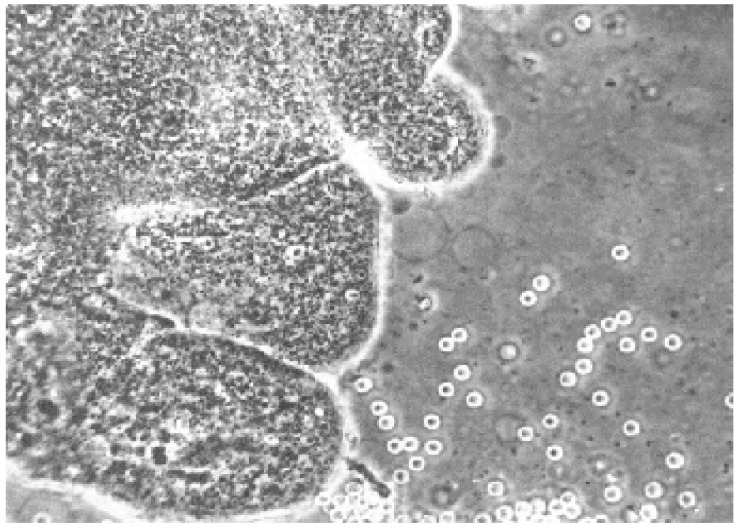
Chorionic villus tissue observed under a dissecting microscope. Source: Authors.

The collected villus tissue samples were promptly transported to the laboratory and examined under a dissecting microscope (Figure 2), following established protocols described in the literature [1,2,3,4,6,8,10,11,12,13,19,20,21,22,23,26]. For cytogenetic analysis, either an overnight short-term incubation (approximately three days) or a standard seven-day culture method was employed. In all cases, fetal heart rate (FHR) was assessed before and after the procedure using M-mode ultrasonography. In a subset of 200 patients, alpha-fetoprotein (AFP) levels were measured 10 min before and 10 min after the CVS procedure to assess the presence of feto-maternal hemorrhage.

Transvaginal color Doppler was utilized to appraise the uteroplacental and fetal vessels velocity waveforms in 400 pregnancies before and after CVS (300 TA-CVS and 100 TC-CVS), with randomized selection. Doppler measurements of the uterine artery, umbilical artery, spiral arteries and middle cerebral artery were conducted 10 min before and 10 min after both transabdominal (TA-CVS) and transcervical chorionic villus sampling (TC-CVS). All assessments were performed using a Voluson 8–10 GE ultrasound system equipped with a 5–9 MHz transvaginal probe. The velocity wave forms were then recorded and the pulsatility index (PI) was calculated automatically [4,6,7,10,12,16,24,25,26,27,30,31,32,33,34,35]. All Rh-negative women received a 50 µg dose of anti-D immunoglobulin following the CVS procedure.

In person follow up was planned with each patient. The information about successful deliveries and neonatological findings has been obtained directly from the relevant hospitals and/or patients, and all data have been computerized and may be accessed promptly via the relevant index or the information displayed herein. Pediatric reports assessing intellectual development five years after delivery were requested from 4400 participants.

Statistical analyses were performed to evaluate differences in fetal outcomes, sampling adequacy and vascular response among the three CVS techniques. A chi-square test for independence was applied to compare fetal loss rates across the TA-CVS, TC-CVS and TV-CVS) groups. Significant associations were followed by pairwise comparisons using Fisher’s exact test with Bonferroni correction for multiple testing. For the analysis of vascular response, paired sample *t*-tests were used to compare pre- and post-procedure pulsatility index (PI). A *p*-value less than 0.05 (two-tailed) was considered statistically significant. Statistical analysis was performed in R, version 4.3.3 (R Foundation for Statistical Computing, Vienna, Austria), using base packages.

## 3. Results

Of the 5500 patients, 850 (15.5%) underwent TC-CVS, 150 (2.7%) underwent TV-CVS, and 4500 (81.8%) underwent TA-CVS. A total of 58.8% of TV-CVS (500 cases), 100% of TV-CVS (150 cases) and 18.9% of TA-CVS (850 cases) were performed between January 2008 and December 2012. From December 2012 onward, TA-CVS became the most frequently used method. The primary indication for CVS was advanced maternal age (≥37 years), accounting for 65% of cases. This is visible in Table 1, alongside the full list of indications. Table 2 presents the distribution of early complications, cytogenetic findings and total fetal loss across 5500 CVS procedures, stratified by method.

In total, 5500 consecutive chorionic villus sampling (CVS) procedures were analyzed, comprising 850 transcervical (TC-CVS), 4500 transabdominal (TA-CVS) and 150 transvaginal (TV-CVS) cases. A total of 850 patients (15.5%) underwent TC-CVS, with spontaneous abortion occurring in 5 cases (0.6%) before 28 weeks of gestation. Among the 4500 patients (81.8%) who received TA-CVS, spontaneous abortion was observed in 8 cases (0.18%). In the TV-CVS group, which included 150 patients (2.7%), 2 spontaneous abortions were recorded (1.3%). The rate of spontaneous abortion was lower in TA-CVS and TC-CVS compared to TV-CVS. These outcomes were compared with a control group of 4777 pregnancies with similar indications for TA-CVS, but without any invasive procedures. In this elective group, the miscarriage rate before 28 weeks was 0.40%, which was higher than the rate observed in the TA-CVS group (0.18%). For TC-CVS, a control group of 875 non-intervention pregnancies showed a miscarriage rate of 1.1% (10 cases), while in the TV-CVS control group of 165 pregnancies, miscarriage occurred in 5 cases (3.5%).

The overall fetal loss rate observed across all procedures was 4.3% (236 out of 5500). When stratified by method, fetal loss occurred in 7.7% of TC-CVS cases (66 out of 850), 3.6% of TA-CVS cases (162 out of 4500) and 5.3% of TV-CVS cases (8 out of 150). A chi-square test for independence was performed to examine whether the method of CVS was associated with a statistically significant difference in fetal loss rates. The analysis demonstrated a significant association between the type of CVS procedure and fetal loss (χ^2^ = 24.56, df = 2, *p* < 0.001), suggesting that the probability of fetal loss is not evenly distributed across the three groups. To further explore which comparisons contributed to the overall significance, pairwise post hoc testing was conducted using Fisher’s exact test with Bonferroni correction for multiple comparisons. The comparison between TC-CVS and TA-CVS revealed a statistically significant difference in fetal loss rates (adjusted *p* = 1.13 × 10^−6^), indicating that TA-CVS is associated with a significantly lower risk of fetal loss compared to TC-CVS. However, no significant differences were observed between TC-CVS and TV-CVS (adjusted *p* = 1.000) or between TA-CVS and TV-CVS (adjusted *p* = 0.791). Taken together, these findings suggest that transabdominal CVS may be the safest method in terms of minimizing fetal loss, particularly when compared to the transcervical approach. The transvaginal method, while less frequently performed in this dataset, did not show statistically significant differences in fetal loss compared to the other two methods.

Chorionic villus sampling was performed in 25% of cases between 10 and 11 weeks of gestation (crown–rump length: 10–16 mm), in another 25% between 11 and 12 weeks (17–26 mm), and in 50% between 12 and 13.6 weeks (27–80 mm). In 96.6% of procedures, a single sampling attempt was sufficient, while in 3.4% of cases, a second attempt was required to obtain an adequate tissue sample. No clear association was observed between gestational age and the success of the first insertion. Adequate tissue weight was generally higher in samples obtained via TC-CVS and TV-CVS compared to TA-CVS (Table 3), and all collected samples were sufficient for diagnostic analysis. Bleeding between the time of sampling and delivery was documented in 34 patients (4.9%), and a placental hematoma measuring 0.5 to 1 mL was noted at the biopsy site in 8 cases (1.4%). Bleeding occurred more frequently after TC-CVS than after TA-CVS or TV-CVS, while peritoneal reaction was observed only following TA-CVS. Infections were recorded in 3 patients (0.4%) after TC-CVS, 2 patients (0.3%) after TV-CVS, and 1 patient (0.02%) after TA-CVS. Among those with post-procedure infection, two pregnancies ended in miscarriage within 2 weeks.

The analysis of sample weight distribution across the three chorionic villus sampling methods revealed statistically significant differences. Among the 5500 procedures, 4500 were transabdominal, 850 transcervical and 150 transvaginal. The weight of the samples was categorized into three groups: <9 mg, 10–20 mg, and >20 mg. Transabdominal CVS yielded the most favorable distribution, with 66.7% of samples falling within the optimal 10–20 mg range, compared to 35.3% for transcervical and 36.7% for transvaginal CVS. Conversely, transcervical and transvaginal approaches were associated with higher proportions of both low-weight (<9 mg: 17.6% and 13.3%, respectively) and high-weight samples (>20 mg: 47.1% and 50.0%, respectively), compared to transabdominal CVS (11.1% and 22.2%, respectively). A Chi-square test of independence confirmed a highly significant association between CVS method and sample weight category (χ^2^ = 350.92, df = 4, *p* < 0.001). This suggests that the transabdominal approach provides more consistent sampling within the desired weight range and may be preferable for achieving optimal sample quality.

Cytogenetic results were obtained in 5480 of 5500 CVS (99.6%) cases. In ten (0.2%) cases tissue was unsuitable for study purposes; however, we have found abnormal cytogenetic results in 220 (4%) of cases. In five of the fourteen cases (35.7%) involving spontaneous abortion after CVS, abnormal cytogenetic findings were detected, indicating that many losses likely reflected underlying fetal chromosomal abnormalities. Separately, in pregnancies with major structural abnormalities on ultrasonography, we performed targeted copy number variant (CNV) analysis in 35 fetuses in line with published recommendations [6,13,14,15,16,28,29,30,33]. In 5 (20.0%) fetuses from this group of patients, we identified pathological CNVs: 22q11.2 microdeletion syndrome; 22q11.2 duplication (in two cases with hydrops fetalis); 10q26–12q26.3–12q21q22 deletion (in a patient of advanced maternal age, 45 years); 5q21.1–q34 deletion (associated with cardiac anomaly); and 46,XX,del(X)(p11.3p22.3) (associated with hygroma colli, hydrothorax, omphalocele and hydrops fetalis). All five CNV-positive pregnancies were electively terminated; none miscarried. Thus, ultrasonographic anomalies warrant CNV testing (20% diagnostic yield in our series), and the cytogenetic abnormalities observed among spontaneous abortions support fetal pathology as an important driver of pregnancy loss.

The total post-procedure fetal loss rate (spontaneous abortion rate and termination of pregnancy) was observed in 236 (4.5%) cases: more specifically, in 66 (7.7%) cases after TC-CVS, in 162 (3.4%) after TA-CVS, and in 8 (5.3%) after TV-CVS. The number of perinatal deaths was zero. Our findings also indicate that the rate of spontaneous abortion following CVS decreases progressively with advancing gestational age at the time of sampling. Specifically, the abortion rate was 0.6% before 11 weeks, 0.4% before 12 weeks, 0.2% before 13 weeks, and 0.16% before 14 weeks. Among younger patients (under 30 years of age), the spontaneous abortion rate remained low, ranging from 1.4% (3 cases) in women under 30 to 4.7% (11 cases) in women aged 40 years or older. In older patients, total fetal loss was more frequent when sampling was performed before 11 weeks; conversely, when CVS was carried out after 13 weeks of gestation, the overall fetal loss rate dropped from 4.5% to 0.15%.

Changes in serum alpha-fetoprotein (AFP) levels were observed in 25 out of 200 patients (12.5%) who had samples taken both before and after CVS. However, no correlation was found between AFP elevation, the amount of chorionic villi aspirated, or the rate of spontaneous abortion following the procedure. Additionally, no notable differences in fetal heart rate (FHR) were detected before and after CVS.

Transvaginal color Doppler was used to investigate the uteroplacental and fetal vessels in 400 pregnancies (300 TA-CVS and 100 TC-CVS) before and after CVS. The main uterine artery, the spiral arteries (located near the CVS site), the umbilical arteries, and the middle cerebral artery were examined. Pulsatility index (PI) measurements were compared before and 10 min after chorionic villus sampling (CVS) in a cohort of 400 participants to evaluate potential vascular responses (Table 4). Paired sample *t*-tests were conducted for five different arteries. No statistically significant changes in PI were observed in the uterine artery (mean before: 2.10 ± 0.55; after: 2.07 ± 0.58; *p* = 0.171), spiral artery (0.60 ± 0.55 vs. 0.59 ± 0.18; *p* = 0.521), or interplacental artery (0.48 ± 0.15 vs. 0.50 ± 0.16; *p* = 0.638). In contrast, a statistically significant decrease in PI was found in the umbilical artery following CVS (2.96 ± 1.42 vs. 2.86 ± 1.55; *p* = 0.032), as well as in the middle cerebral artery (1.88 ± 0.55 vs. 1.75 ± 0.60; *p* < 0.001) (Table 4). These findings suggest that while most arterial PI values remain stable after CVS, the umbilical and middle cerebral arteries may exhibit subtle but measurable hemodynamic changes shortly after the procedure.

Follow-up was possible in all the cases included in this study. We have further detected fetal anomalies in 55 cases (1%): hygroma colli multiloculare in 11 cases, cardiac anomalies in 10, genitourinary abnormalities in 9, central nervous system anomalies and universal hydrops each in 6 cases, abdominal wall defects in 5, and facial clefting in 1 case (Table 5). However, no serious limb abnormalities were observed in any of the cases. Pediatric reports on intellectual development five years after delivery were requested from 4400 participants, and no cases of intellectual disability were identified.

## 4. Discussion

This large, prospective study of 5500 singleton pregnancies represents one of the most comprehensive evaluations of first-trimester CVS to date, directly comparing the TA-CVS, TC-CVS and TV-CVS approaches. Beyond the general recommendation favoring the abdominal approach, our study adds a single-operator perspective with high-volume series minimizing learning-curve effects; route-specific control cohorts from the same referral stream and gestational windows to estimate excess risk over background and to separate procedure-related miscarriage from overall loss (including elective terminations after abnormal results); substantial TC-CVS representation from earlier years within uniform laboratory workflows; and integrated reporting of targeted CNV yield alongside 5-year pediatric-neurology outcomes. These elements are rarely presented together.

In our study, TA-CVS showed the most favorable safety profile, with the lowest rates of spontaneous abortion, infection and early complications. Sample adequacy was high across all methods, though TA-CVS consistently yielded samples within the optimal weight range. Miscarriage rates decreased with advancing gestational age and were lowest in younger patients. Doppler ultrasound revealed no major hemodynamic disturbances, except for slight reductions in umbilical and middle cerebral artery PI. Cytogenetic abnormalities were detected in 4% of cases, and pathogenic CNVs were identified in 20% of fetuses with structural anomalies. No serious limb defects or intellectual disabilities were observed at follow-up. Our findings support the preferential use of TA-CVS as a first-line approach, especially in centers with high procedural volume and expertise. The data also underscore the importance of careful gestational timing and CNV testing in cases with sonographic anomalies.

CVS is indeed a valuable diagnostic option for women at elevated genetic risk, habitually performed between the 10th and 14th weeks of gestation. When conducted by experienced practitioners, CVS is associated with a low incidence of complications. Several comparative studies have evaluated the safety profiles of CVS versus amniocentesis in pregnancies confirmed as viable during first-trimester ultrasound [1,3,4,5,6,7,8,9,10,11,12,13,15,16,17,18]. Findings suggest that the risk of pregnancy loss may be 1–2% higher, or potentially more, following CVS than amniocentesis [1,3,4,5,6,7,8,9,10,11,12,13,15,16,17,18]. A study by Crane et al. [11] reported no significant difference in miscarriage rates between the two procedures. Currently, the two predominant methods for CVS are TA-CVS and TC-CVS. A large retrospective Cochrane cohort study published examined outcomes from 4862 CVS procedures, of which 2833 were performed transcervically—using either forceps (*n* = 1787) or cannulas (*n* = 1046). The overall pregnancy loss rate was 1.4% for TC-CVS and 1.0% for TA-CVS. Notably, within the TC-CVS group, the loss rate was markedly lower at 0.27% when forceps were used, compared to 3.12% with cannula-based procedures, indicating a possible safety benefit associated with forceps [5,6,7,8,9,28,29,30,31,32,33,34,35,36,37,38].

In contrast, this 15-year prospective study found lower overall complication rates across all methods. Specifically, the spontaneous abortion rate following TC-CVS was 0.6%, substantially lower than the 1.4% reported in the Cochrane study. Similarly, the TA-CVS loss rate in our cohort was 0.18%, compared to 1.0% in the previous report. The TV-CVS method, used in a smaller number of cases (*n* = 150), was associated with a fetal loss rate of 1.3%. These differences may be attributed to methodological variations, operator experience and the consistent application of standardized techniques in our setting. Our findings further support the relative safety of TA-CVS and suggest that when TC-CVS is performed with precision, its complication rates may be lower than previously reported in broader datasets.

Procedure-related pregnancy loss was also considered in relation to operator experience, with approximately 1000 invasive prenatal diagnostic procedures performed annually at our center [5,6,7,8,9,28,29,30,31,32,33,34,35,36,37,39,40]. Miscarriage rates were compared with those in a control group of pregnancies that did not undergo any invasive procedures. In this elective group, miscarriage occurred in 20 pregnancies (0.4%), which is the same rate observed in the TA-CVS group. According to Brambati et al. [35], fetal loss before 28 weeks of gestation in pregnancies intended to continue was 2.58%, with higher rates observed in older maternal age groups. In our comparison of control pregnancies for TC-CVS, 875 elective cases were analyzed, with miscarriage occurring in 10 pregnancies (1.1%). In the control group for TV-CVS, consisting of 165 elective pregnancies, 5 miscarriages were recorded (3.5%). The miscarriage rate was lower in the TA-CVS group. Over the last five years, TA-CVS was consistently performed at 13 weeks of gestation by a single operator (M.P.) in the study setting. In procedures performed by experienced operators in specialized centers, no significant increase in miscarriage risk are observed, suggesting that the actual risk of miscarriage is lower than often reported [6,8,10,13,14,15,16].

In interpreting pregnancy-loss outcomes, the higher miscarriage rate observed in some control cohorts (most notably relative to TA-CVS) likely reflects case-mix and outcome-ascertainment dynamics rather than an adverse effect of CVS. In the CVS groups, serious chromosomal problems were found early and many of those pregnancies were electively ended, which means later miscarriages did not occur or were not counted. In controls, similar high-risk pregnancies were not tested invasively and some ended later as miscarriages, which makes the control rate look higher. Additionally, route-linked differences in timing and indication mix (e.g., TA procedures more often performed within our preferred 12–13 + 6 week window) and smaller, higher-risk control subsets for TC and TV can yield greater variation between control groups than between each intervention-control pair.

Mosaicisms and other aneuploidies confined to placental tissue (whether limited to the trophoblast or mesenchymal components) have been previously documented [2,4,5,13,14,19,20,21,22,23,24,25,30]. In first-trimester short-term chorionic villus cultures, mosaicisms are estimated to occur in approximately 2–3% of cases, though many are not confirmed in subsequent testing. This raises potential challenges for interpreting such findings in ongoing pregnancies. To improve diagnostic accuracy, follow-up studies using long-term villus cultures and fetal karyotyping are recommended. In our cohort, mosaicism was identified in 55 cases (1%), with confirmatory fetal blood sampling via cordocentesis conducted in all. Of these, true fetal mosaicism was confirmed in 25 cases (45.5%). Also, it has to be emphasized that, in interpreting cytogenetic yields, the higher abnormality rate in the TC-CVS group (7.4% vs. 3.5% for TA-CVS) most plausibly reflects temporal and case-mix differences (TC-CVS predominated in earlier years when higher-risk indications were more frequent) rather than procedural sensitivity, given uniform laboratory workflows and the fact that the sampling route does not affect placental genotype.

The observations in this paper can be compared with the recent study by Liu et al., who found that chromosomal abnormalities in fetal tissue are the main cause of spontaneous abortion [41]. The risk of such abnormalities after miscarriage was higher with maternal age over 35 years, gestational age under 12 weeks, and with ≤4 prior pregnancy losses [41]. Findings can also be linked to the 30-year Croatian cystic hygroma cohort, which also showed how early sonographic markers can strongly predict chromosomal pathology and merit comprehensive genetics [42]. This supports a pathway in which route-appropriate CVS provides reliable tissue for definitive cytogenetics when a certain abnormality is detected, while also clarifying prognosis.

Elevated maternal serum AFP levels following CVS were observed in 16% of cases. However, no correlation was found between AFP increase and the occurrence of placental hematoma, Doppler findings in fetal or maternal circulation or spontaneous abortion post-procedure. Transient feto-maternal transfusion was evident in most cases, as indicated by changes in maternal AFP levels before and after sampling. The extent of fetal hemorrhage was significantly related to the weight of chorionic tissue aspirated. In all CVS procedures, the collected villous tissue volume was sufficient for cytogenetic analysis. Our laboratory required at least 5 mg of tissue to conduct reliable chromosome studies [19,20,34]; consequently, clinically significant feto-maternal hemorrhage was detected in rare instances only.

Fetal hemorrhage may influence embryonic organogenesis by inducing circulatory collapse and tissue hypoxia. In cases of severe vascular disruption, this can initiate a cascade of hypoperfusion events leading to tissue damage. In a systematic review by Salamon et al. [13], out of 2943 screened citations, seven studies focused on CVS and 12 on the rate of limb abnormalities. These were often linked with oro-mandibular hypogenesis, most notably in procedures performed during the earliest stages of limb development. Their findings support the hypothesis that very early CVS may contribute etiologically to such defects. A slight increase in limb abnormalities was noted when CVS was conducted before 9 weeks of gestation. While the risk may be related to procedural timing, it is unclear whether modifications to technique can fully mitigate this risk [4,5,6,12,13,14,27,34,35,38]. In our cohort, no severe limb malformations were observed, likely because 50% of CVS procedures were performed after 12 weeks of gestation.

This study has several limitations that should be considered when interpreting the findings. First of all, it was conducted at a single high-volume center, and all procedures were performed by one operator, which limits external validity and may introduce operator-specific effects. In other words, our findings are most relevant to settings with similar operator experience, timing policies, route-selection criteria, counseling and laboratory workflows; extrapolation to lower-volume or differently organized services should be cautious, as case-mix, referral timing and operator proficiency can shift both cytogenetic yield and observed loss rates. Regarding other limitations, in our research approach time-stratified analyses were not performed; however, while the study spans 15 years, calendar time is unlikely to confound outcomes due to the operator’s long-standing proficiency and consistent protocols. Route selection was driven by clinical pragmatics (e.g., placental location), thus residual confounding by indication is likely. Furthermore, the analyses were largely unadjusted; the absence of multivariable modeling means differences in maternal and gestational characteristics, placenta site, number of passes or sample weight could account for some outcome differences. Then, hemodynamic (Doppler) and AFP assessments were performed only in subsets with short immediate follow-up, which may miss delayed or transient effects and precludes conclusions for the transvaginal route. Microarray/CNV testing was restricted to fetuses with major structural anomalies, limiting generalizability of the reported yield. Because control assignment reflected patient choice rather than randomization, residual self-selection and unmeasured confounding (e.g., risk aversion, nuanced case-mix) cannot be fully excluded despite comparable eligibility and referral timing. Finally, inclusion was limited to singleton pregnancies in a standardized, specialized setting, so results may not extrapolate to multiple gestations, earlier gestational ages, or to lower-volume centers.

## 5. Conclusions

Our findings indicate that CVS did not result in measurable alterations to uteroplacental blood flow, as assessed by Doppler ultrasound before and after the procedure. This suggests that subsequent fetal losses were unlikely to be caused by vascular disruption within the uteroplacental unit. Among the three approaches evaluated, TA-CVS demonstrated the lowest fetal loss rate, potentially due to the lower quantity of chorionic tissue aspirated, which in many cases was below 20 mg. Despite this smaller volume, sample adequacy for cytogenetic analysis was consistently achieved, and the ability to obtain sufficient yet minimally invasive samples may contribute to the safety profile of TA-CVS.

In any case, we believe adequate sample size is critical for ensuring diagnostic accuracy, especially in cases where chromosomal anomalies or CNVs are suspected. However, there remains a paucity of data on how specific procedural or patient-related factors (for example sample weight or placental location) influence the risk of spontaneous abortion after CVS. TA-CVS in our practice was associated with lower observed pregnancy loss, but definitive comparisons of procedural safety require prospective, standardized multicentre and prospective studies (with standardized protocols) that distinguish procedure-related miscarriage from losses due to selective termination, in order to clarify the relationship between these variables and pregnancy outcomes. In our cohort, the absence of vascular complications, coupled with high diagnostic yield, supports the use of TA-CVS as the preferred approach in early prenatal testing, provided it is performed by experienced operators in specialized settings.

## Figures and Tables

**Table 1:** Indications for first-trimester chorionic villus sampling in 5500 cases. *N* = number of cases; % = percentage of total cohort; NIPT = non-invasive prenatal testing.

	*N*	%
Maternal age (≥37 years)	3575	65.0
Potentially abnormal ultrasound or NIPT finding	1375	25.0
Previous child diagnosed with a chromosomal disorder	110	2.0
Known parental balanced chromosomal translocations	110	2.0
Risk of metabolic disease (monogenic)	80	1.5
Risk of thalassemia or haemoglobinopathy	80	1.5
Fetal sex determination (including cases with risk of X-linked disorders)	40	0.7
History of drug or radiation exposure	45	0.8
Family history of intellectual disability	55	1.0
Other or unspecified concerns (e.g., maternal anxiety)	30	0.5

**Table 2:** Clinical outcomes and early complications observed across all chorionic villus sampling procedures performed in the study. TC-CVS = transcervical chorionic villus sampling; TA-CVS = transabdominal chorionic villus sampling; TV-CVS = transvaginal chorionic villus sampling.

	TC-CVS		TA-CVS		TV-CVS		Total	
	*N*	%	*N*	%	*N*	%	*N*	%
Total number	850	15.5	4500	81.8	150	2.7	5500	100.0
Early complications								
Bleeding	34	4.0	45	1.0	15	10.0	94	1.7
Infection	4	0.4	2	0.4	2	3.0	0.2	0.145
Spontaneous abortion	5	0.6	8	0.18	2	1.3	14	0.66
Abnormal cytogenetic results	61	7.1	154	3.4	5	3.3	220	4.0
Selective abortion	58	6.8	150	3.3	5	3.3	213	3.9
Total fetal loss	63	7.4	158	3.5	7	5.3	228	4.1

**Table 3:** Amount of chorionic villus tissue obtained during CVS procedures. CVS = chorionic villus sampling.

CSV Method	<9 mg		10–20 mg		>20 mg	
	*N*	%	*N*	%	*N*	%
Transabdominal CVS	500	11.1	3000	66.7	1000	22.2
Transcervical CVS	150	17.6	300	35.3	400	47.1
Transvaginal CVS	20	13.3	55	36.7	75	50.0

**Table 4:** Pulsatility index (PI) values in the main uterine artery, spiral arteries, intraplacental arteries, umbilical artery, and middle cerebral artery before and after chorionic villus sampling (CVS).

*N* = 400	PI 10 min		PI 10 min	
	Before CVS		After CVS	
	Mean	1 SD	Mean	1 SD
Uterine artery	2.10	0.55	2.07	0.58 *p* = 0.171
Spiral artery	0.60	0.55	0.59	0.18 *p* = 0.521
Intraplacentar artery	0.48	0.15	0.50	0.16 *p* = 0.638
Umbilical artery	2.96	1.42	2.86	1.55 *p* = 0.032
Middle cerebral artery	1.88	0.55	1.75	0.60 *p* < 0.001

**Table 5:** Malformations detected in utero or at birth in 5500 cases following chorionic villus sampling. *N* = number of cases; mg = milligrams (amount of chorionic tissue obtained).

Fetal Malformation (*N*)	Amount of Chorionic Tissue (mg)
Anencephaly 2	20
Hydrocephalus 2	25
Spina bifida 2	30
Cleft-lip palate 1	20
Cardiac anomaly 10	30
Esophageal atresia 1	25
Diaphragmatic hernia 2	20
Hygroma colli multilocularae 11	30
Omphalocele 5	25
Renal agenesis 3	15
Cystic kidneys 6	20
Hydrops universalis 6	25
Thanatophoric dysplasia 1	15
Polydactyly 3	20
Limb reduction0	20
Total 55 (1%)	20.3 ± 9.7 mg

## Data Availability

The data presented in this study are available on request from the corresponding author due to privacy concerns.
